# Supplementary material for: Evaluation of Ascophyllum nodosum extract supplementation on feed degradability, ruminal fermentation and methane production using the rumen simulation technique (RUSITEC)
Source: PLoS One. 2026 Mar 25;21(3):e0344019. doi: 10.1371/journal.pone.0344019 (PMC13016304; doi:10.1371/journal.pone.0344019)
Supplement: Attachment — Submitted filename: PONE-D-25-49338_reviewer_AS.pdf [file pone.0344019.s002.pdf]

PLOS ONE

Evaluating the effect of an Ascophyllum nodosum extract on diet degradability, ruminal fermentation and methane production using the rumen simulation technique (RUSITEC)

--Manuscript Draft--

**Manuscript Number:**	PONE-D-25-49338
**Article Type:**	Research Article
**Full Title:**	Evaluating the effect of an Ascophyllum nodosum extract on diet degradability, ruminal fermentation and methane production using the rumen simulation technique (RUSITEC)
**Short Title:**	The effect of an Ascophyllum nodosum extract on methane production in the RUSITEC
**Corresponding Author:**	Stafford Vigors, PhD<br>University College Dublin<br>Dublin, Dublin IRELAND
**Keywords:**	Ascophyllum nodosum<br>Methane<br>Phlorotannin's<br>Rumen fermentation<br>RUSITEC
**Abstract:**	This study investigated the effects of liquid Ascophyllum nodosum (ASC) extracts on diet degradability, rumen fermentation, total gas and methane (CH4) production using the rumen simulation technique (RUSITEC). Two experiments were conducted: Experiment 1 assessed a grass-based diet with an ASC extract included at 0.1%, 0.2% and 0.3% of dry matter (DM), while Experiment 2 evaluated a grass silage-based diet with an ASC extract included at 0.3%, 0.4% and 0.5% of DM. Both diets included a control treatment (CTR; no seaweed extract). In Experiment 1, the 0.3% treatment reduced CH4 relative to CTR, primarily by decreasing nutrient degradation parameters and total volatile fatty acids. This resulted in lower production of total gas, CH4 mmol/d and CH4 mmol/g of digestible organic matter (DOM). Metabolic hydrogen produced and incorporated were also reduced at 0.3% relative to CTR. In Experiment 2, the 0.5% treatment decreased DM and organic matter degradation, with no effects on crude protein or neutral detergent fibre degradation, or any rumen fermentation parameters. Despite this, in Experiment 2, all ASC extract treatments reduced the production of total gas, CH4 mmol/d and CH4 mmol/g DOM relative to CTR. There was no impact on metabolic hydrogen produced or incorporated, however metabolic hydrogen recovery was reduced across all ASC extract treatments, suggesting other mechanisms were at play redirecting hydrogen away from methanogenesis. These findings highlight the potential of ASC extracts to reduce CH4 production through diet-specific mechanisms and support sustainable ruminant production systems.
**Order of Authors:**	Stafford Vigors, PhD
	Aaron Casey
	Tommy Boland
	Alan Kelly
	Zoe McKay
	Maria Markiewicz-Keszycka
	Armin Mirzapour-Koudash
	Marco Garcia-Vaquero
	Sivaprakash Balakrishnan
	Kieran J. Guinan
	John T. O'Sullivan

Powered by Editorial Manager® and ProduXion Manager® from Aries Systems Corporation

Additional Information:	
**Question**	**Response**
**Financial Disclosure**<br><br>Enter a financial disclosure statement that describes the sources of funding for the work included in this submission. Review the submission guidelines for detailed requirements. View published research articles from *PLOS ONE* for specific examples.<br><br>This statement is required for submission and **will appear in the published article** if the submission is accepted. Please make sure it is accurate.<br><br>**Funded studies**<br>Enter a statement with the following details:<br>• Initials of the authors who received each award<br>• Grant numbers awarded to each author<br>• The full name of each funder<br>• URL of each funder website<br>• Did the sponsors or funders play any role in the study design, data collection and analysis, decision to publish, or preparation of the manuscript?<br><br>Did you receive funding for this work?	The author(s) received no specific funding for this work.
**Competing Interests**<br><br>Use the instructions below to enter a competing interest statement for this submission. On behalf of all authors, disclose any competing interests that could be perceived to bias this work—acknowledging all financial support and any other relevant financial or non-financial competing interests.<br><br>This statement is required for submission and **will appear in the published article** if the submission is accepted. Please make sure it is accurate and that any funding sources listed in your Funding Information	The authors have declared that no competing interests exist.

later in the submission form are also declared in your Financial Disclosure statement.<br><br>View published research articles from *PLOS ONE* for specific examples.<br><br>**NO authors have competing interests**<br><br>Enter: *The authors have declared that no competing interests exist.*<br><br>**Authors with competing interests**<br><br>Enter competing interest details beginning with this statement:<br><br>*I have read the journal's policy and the authors of this manuscript have the following competing interests: [insert competing interests here]*<br><br>* typeset	
**Ethics Statement**<br><br>Enter an ethics statement for this submission. This statement is required if the study involved:<br><br>• Human participants<br>• Human specimens or tissue<br>• Vertebrate animals or cephalopods<br>• Vertebrate embryos or tissues<br>• Field research<br><br>Write "N/A" if the submission does not require an ethics statement.<br><br>General guidance is provided below. Consult the submission guidelines for detailed instructions. **Make sure that all information entered here is included in the Methods section of the manuscript.**	N/A

**Format for specific study types**<br><br>**Human Subject Research (involving human participants and/or tissue)**<br>• Give the name of the institutional review board or ethics committee that approved the study<br>• Include the approval number and/or a statement indicating approval of this research<br>• Indicate the form of consent obtained (written/oral) or the reason that consent was not obtained (e.g. the data were analyzed anonymously)<br><br>**Animal Research (involving vertebrate animals, embryos or tissues)**<br>• Provide the name of the Institutional Animal Care and Use Committee (IACUC) or other relevant ethics board that reviewed the study protocol, and indicate whether they approved this research or granted a formal waiver of ethical approval<br>• Include an approval number if one was obtained<br>• If the study involved *non-human primates*, add *additional details* about animal welfare and steps taken to ameliorate suffering<br>• If anesthesia, euthanasia, or any kind of animal sacrifice is part of the study, include briefly which substances and/or methods were applied<br><br>**Field Research**<br><br>Include the following details if this study involves the collection of plant, animal, or other materials from a natural setting:<br>• Field permit number<br>• Name of the institution or relevant body that granted permission	
**Data Availability**<br><br>Authors are required to make all data underlying the findings described fully available, without restriction, and from the time of publication. PLOS allows rare exceptions to address legal and ethical concerns. See the PLOS Data Policy and FAQ for detailed information.	No - some restrictions will apply

Powered by Editorial Manager® and ProduXion Manager® from Aries Systems Corporation

A Data Availability Statement describing where the data can be found is required at submission. Your answers to this question constitute the Data Availability Statement and **will be published in the article**, if accepted.<br><br>**Important:** Stating 'data available on request from the author' is not sufficient. If your data are only available upon request, select 'No' for the first question and explain your exceptional situation in the text box.<br><br>Do the authors confirm that all data underlying the findings described in their manuscript are fully available without restriction?	
**Describe where the data may be found in full sentences. If you are copying our sample text, replace any instances of XXX with the appropriate details.**<br><br>• If the data are **held or will be held in a public repository**, include URLs, accession numbers or DOIs. If this information will only be available after acceptance, indicate this by ticking the box below. For example: *All XXX files are available from the XXX database (accession number(s) XXX, XXX.).*<br>• If the data are all contained **within the manuscript and/or Supporting Information files**, enter the following: *All relevant data are within the manuscript and its Supporting Information files.*<br>• If neither of these applies but you are able to provide **details of access elsewhere**, with or without limitations, please do so. For example:<br><br>*Data cannot be shared publicly because of [XXX]. Data are available from the XXX Institutional Data Access / Ethics Committee (contact via XXX) for researchers who meet the criteria for access to confidential data.*<br><br>*The data underlying the results presented in the study are available from (include the name of the third party*	All relevant data are within the manuscript and its Supporting Information files. Raw data underlying the results are owned in part by BioAtlantis Ltd and cannot be shared publicly due to third-party restrictions. Data may be made available from corresponding author contact details for researchers who meet the criteria for access to confidential data.

Powered by Editorial Manager® and ProduXion Manager® from Aries Systems Corporation

and contact information or URL).<br>• This text is appropriate if the data are owned by a third party and authors do not have permission to share the data.<br><br>* typeset	
Additional data availability information:	Tick here if the URLs/accession numbers/DOIs will be available only after acceptance of the manuscript for publication so that we can ensure their inclusion before publication.

Powered by Editorial Manager® and ProduXion Manager® from Aries Systems Corporation

Click here to access/download;Manuscript;RUSITEC - PloS ONE.docx

~~Evaluating the effect of~~ an *Ascophyllum nodosum* extract on diet degradability, ruminal fermentation and methane production using the rumen simulation technique (RUSITEC)

Aaron Casey[1], Tommy Boland[1], Alan Kelly[1], Zoe McKay[1], Maria Markiewicz-Keszycka[1], Armin Mirzapour-Koudash[1], Marco Garcia-Vaquero[1], Sivaprakash Balakrishnan[2], Kieran J. Guinan[2], John T. O'Sullivan[2] and Stafford Vigors[1*]

1 School of Agriculture and Food Science, University College Dublin, Belfield, Dublin 4, Ireland, **2** BioAtlantis Ltd, Clash Industrial Estate, Tralee, Co. Kerry, Ireland

*Stafford.vigors1@ucd.ie

Abstract

This study investigated the effects of liquid *Ascophyllum nodosum* (ASC) extracts on diet degradability, rumen fermentation, total gas and methane (CH_4) production using the rumen simulation technique (RUSITEC). Two experiments were conducted: Experiment 1 assessed a grass-based diet with an ASC extract included at 0.1%, 0.2% and 0.3% of dry matter (DM), while Experiment 2 evaluated a grass silage-based diet with an ASC extract included at 0.3%, 0.4% and 0.5% of DM. Both diets included a control treatment (CTR; no seaweed extract). In Experiment 1, the 0.3% treatment reduced CH_4 relative to CTR, primarily by decreasing nutrient degradation parameters and total volatile fatty acids. This resulted in lower production of total gas, CH_4 mmol/d and CH_4 mmol/g of digestible organic matter (DOM). Metabolic hydrogen produced and incorporated were also reduced at 0.3% relative to CTR. In Experiment 2, the 0.5% treatment decreased DM and organic matter degradation, with no effects on crude protein or neutral detergent fibre degradation, or any rumen fermentation parameters. Despite this, in Experiment 2, all ASC extract treatments reduced the production of total gas, CH_4 mmol/d and CH_4 mmol/g DOM relative to CTR. There was no impact on metabolic hydrogen produced or incorporated, however metabolic hydrogen recovery was reduced across all ASC extract treatments, suggesting other mechanisms were at play redirecting hydrogen away from methanogenesis. These findings highlight the potential of ASC extracts to reduce CH_4 production through diet-specific mechanisms and support sustainable ruminant production systems.

Introduction

Agriculture is responsible for the production of 37.8% of Irish greenhouse gas (GHG) emissions, with methane (CH_4) from ruminal fermentation and manure management contributing up to 72.1% of agricultural emissions [1]. Through ruminal fermentation, feed is degraded by microorganisms to produce volatile fatty acids (VFA), hydrogen (H_2) and carbon dioxide (CO_2). The VFA's are absorbed mainly through the rumen wall and provide energy to the animal, however, H_2 is toxic to the rumen environment, and therefore must be removed to ensure the fermentation process continues [2]. Methanogenesis is considered the main metabolic hydrogen ([H]) sink in the rumen whereby methanogenic archaea reduce CO_2 to CH_4 by utilizing H_2 [3, 4], causing an energy loss of 2-12% of gross energy intake [5]. The European Green Deal aims to reduce GHG emissions by 55% by 2030 compared to 1990 levels [6]. Strategies to achieve these targets include exploring anti-methanogenic feed additives that specifically target methanogenesis and reduce CH_4 emissions. Feed additives can reduce CH_4 by inhibiting H_2 production, providing an alternative H sink, or suppressing methanogenic archaea, with diet and microbiome modifications serving as potential CH_4 mitigation tools [7-9].

Previous research has explored dietary feed additives, including ionophores, chemical compounds, essential oils, and plant secondary compounds, to mitigate CH_4 emissions [10-12]. Seaweeds have promise for CH_4 mitigation [13], particularly red seaweed species like *Asparagopsis taxiformis* [14, 15]. However, the non-native status of this seaweed in Ireland and the environmental concerns related to the halogenated compound bromoform require the exploration of alternative seaweed solutions. One such species that does not contain bromoform is the brown seaweed *Ascophyllum nodosum* (ASC), which is widely distributed along the shorelines of western Europe. *Ascophyllum nodosum* exhibits promising anti-methanogenic

effects, attributed to the presence of phlorotannin's (PT). Phlorotannin's are similar to terrestrial tannins and have the ability to bind to proteins and carbohydrates [16]. They are reported to inhibit protozoal populations and reduce the activity of fibre-degrading bacteria, which can subsequently decrease methanogenesis [17]. Phlorotannin's are also reported to exert strong anti-microbial activity [18]. *In vitro* studies on dried ASC supplementation have reported reductions in total gas production (TGP), CH_4 [19], nitrogen (N) degradation [20], and ammonia-N concentration in rumen fluid [21].

While many seaweed species are often used in an unprocessed form in animal feeds, the effects of a targeted extraction of potential bioactive components on nutrient utilization and methanogenesis remain relatively unknown. To address this, Wang [18] tested different PT concentrations from ASC in an *in vitro* batch culture system, revealing that PT readily formed complexes with proteins, leading to a linear reduction in fermentation and CH_4 production. However, batch cultures face challenges in maintaining a stable fermentation over multiple days due to the build-up of fermentation products [20]. The rumen simulation technique (RUSITEC), designed by Czerkawski and Breckenridge [22], offers a more effective alternative by allowing for prolonged and consistent *in vitro* fermentation, making it well-suited for screening anti-methanogenic feed additives before conducting costly, time-consuming, and labour-intensive *in vivo* studies [23].

The novelty of this study was to test liquid ASC extracts *in vitro* that could potentially pave the way for future inclusion *in vivo* via water-based delivery. The hypothesis of this study was that supplementing liquid ASC extracts, rich in PT's, in the RUSITEC system would reduce TGP and CH_4 production, thereby identifying their potential for effective *in vivo* CH_4 mitigation. The objective of this study was to evaluate the effects of an ASC extract on *in vitro* nutrient degradation, rumen fermentation and CH_4 production to determine the inclusion level with the strongest anti-methanogenic effect for future application in animal studies.

Materials and Methods

Experimental licensing

This experiment was conducted at UCD Lyons Research Farm, Celbridge, Co. Kildare, Ireland, W23 ENY2 (53^{0} $17^{'}$ $56^{''}$ N, 6^{0} $32^{'}$ $18^{''}$ W). Rumen inoculum during Experiment 2 was harvested from four rumen cannulated Aberdeen Angus x Friesian heifers at Teagasc Grange Animal and Bioscience Research Department, Co. Meath. Animals utilised for the collection of rumen inoculum were cared for in accordance with the Teagasc Animal Ethics Committee. All procedures were approved by the Health Product Regulatory Authority (AE19132/P113), conducted under the European Directive 2010/63/EU and S.I. No. 543 of 2012.

Experimental design and apparatus

The study consisted of two separate complete randomized design 17-d experiments, the initial 10 days dedicated to microbial adaptation and fermentation stabilisation, as outlined by Jaurena [24], and the remaining 7 days for data collection and sampling. Each experiment contained two RUSITEC systems with six vessels per system (Sanshin Industrial Co. Ltd, Yokohama, Japan), used to simulate the rumen environment *in vitro*. Each vessel had a nominal volume of 800 ml and the general incubation procedure was carried out as described by Czerkawski and Breckenridge [22].

Diets and treatments

Different basal diets were employed for each experiment, with all vessels receiving 20 g dry matter (DM) of feed components—comprising of two separate nylon feed bags (100 - µm pore size; 5 x 10 cm – concentrate; 10 x 20 cm – forage; ANKOM ^{TM} Technology, Macedon, NY, USA); one forage bag containing 16 g of forage DM and one concentrate bag containing 4 g of concentrate DM. The forage component of the basal diet offered to the donor animals at

the time of rumen inoculum collection was subsequently then utilized as the forage component for each respective *in vitro* experiment. This approach aimed to minimise time required for microbial adaptation to the experimental diets. For Experiment 1, a perennial ryegrass (*Lolium perenne*) based pasture was utilised, for Experiment 2, a perennial ryegrass-based grass silage was utilized. The feed bags were provided on an 80:20 F:C DM basis. Prior to inclusion in the vessels, both concentrate and forage components underwent a drying process in a forced air oven at 55°C for 48 h. The concentrate feed component was ground through a 1 mm sieve, and the forage component was chopped to 1-2 cm in length using a bowl chopper. The chemical composition of the forages and concentrate is presented in **Table 1**.

Table 1. Chemical composition (g/kg DM unless otherwise stated) of forage and concentrate for Experiment 1 and 2.

Parameter	Experiment 1		Experiment 2	
	Perennial ryegrass pasture	Concentrate	Grass Silage	Concentrate
**DM (g/kg)**	216	894	532	892
**Ash**	78.7	53.7	8.6.0	46.9
**CP**	129	159	82.6	115
**NDF**	379	199	509	186
**ADF**	196	75.1	303	93.8
**OM**	921	946	914	951
**Starch**	.	498	.	448
**GE (MJ/ kg DM)**	17.5	17.5	17.1	17.0
**EE**	30.5	26.3	11.7	19.3

DM, dry matter; CP, crude protein; NDF, neutral detergent fibre; ADF, acid detergent fibre; OM, organic matter; GE, gross energy; EE, ether extract.

In Experiment 1, rumen inoculum was obtained post slaughter from three rumens in an abattoir, where all animals had been offered fresh grass and concentrate prior to slaughter. In

Experiment 2, rumen inoculum was obtained from four ruminally cannulated Aberdeen Angus x Holstein Friesian heifers before the morning feeding at 0800 h, where all animals had been offered grass silage and concentrate.

The seaweed extracts were manufactured by BioAtlantis Ltd. (Clash Industrial Estate, Tralee, Co. Kerry, Ireland) using the brown seaweed *Ascophyllum nodosum*, which was hand harvested in Ireland during the winter season. The process involved aqueous extraction under conditions of high temperature and pH, followed by pH adjustments, filtration and evaporation to reach approximate 50% w/v levels. The extract typically contains 12-16% inorganic matter and 25-30%, organic matter (OM), the latter including approximately 1.5-2.5% mannitol and 7-10% PT on a DM basis, in addition to alginic acid polymers, fucose and glucan polymers. Extracts differed slightly in chemical composition between experiments, with PT concentrations of 163.2 mg PGE/g in Experiment 1 and 142.5 mg PGE/g in Experiment 2.

In Experiment 1, treatments consisted of three seaweed extract inclusion rates of 20 mg/ 20g DM (**0.1%**), 40 mg/ 20g DM (**0.2%**) and 60 mg/ 20g DM (**0.3%**). In Experiment 2, the extract was supplied at 60 mg/ 20g DM (**0.3%**), 80 mg/ 20g DM (**0.4%**) and 100 mg/ 20g DM (**0.5%**). Both experiments included a control diet which received no seaweed extract (**CTR**). Treatments were replicated three times and randomly allocated to each vessel. The chemical composition of the seaweed extract is included in **Table 2**.

Table 2. Chemical composition of *Ascophyllum nodosum* extract for Experiment 1 and 2. Figures presented are mean values ± standard error.

Parameter	Experiment 1	Experiment 2
**DM (g/kg)**	399 ± 0.3	415 ± 0.2
**Ash (g/kg)**	163 ± 1.1	157 ± 0.9
**Total tannin content (mg ChE/g)**	6.60 ± 0.03	6.53 ± 0.05
**Total soluble carbohydrates (mg GlcE/g)**	10.8 ± 0.4	20.3 ± 0.5
**Total phenolic content (mg GAE/g)**	272 ± 0.0	202 ± 0.9

**Total phlorotannin content (mg PGE/g)**	163 ± 0.2	142 ± 0.7

Experimental procedure

On d-0, rumen inoculum (fluid and digesta) was collected, strained through four layers of cheesecloth, pooled, flushed with CO_2, and promptly incubated at 39°C. Within 60 min of collection, the inoculated mixture was transferred to the RUSITEC vessels. Each vessel received 450 ml of rumen inoculum and 350 ml of artificial saliva [25]. Dietary treatments were introduced in nylon bags (100-μm pore size), including 70 g of rumen solid digesta, a second bag containing the 16 g forage DM, and a third bag containing 4 g of concentrate DM.

Artificial saliva, prepared daily, was continuously infused at a rate of 640 ml/d (dilution rate of 3.33%/h) using a peristaltic pump to prevent the washout of rumen microbes. Effluent and fermentation gasses from each vessel were collected into effluent bottles and gas collection bags, respectively. After 24 hours at 0900 h, each vessel was opened, and two of the initial three bags containing rumen digesta solids, along with the bag containing the concentrate, were removed, squeezed, and washed in 50 ml of artificial saliva. Liquid ASC extracts were pipetted into the vessel daily at the designated dosage rate on a single occasion. The liquid fractions of the washings in artificial saliva were returned to the vessels, and two new nylon bags, containing 16 g of forage DM and 4 g of concentrate DM, were inserted. On subsequent days, the nylon bag, containing the forage that had been in the vessel for 48 h was replaced with a new forage bag, and the nylon bag containing the concentrate, which had been in the vessel for 24 h, was similarly replaced.

Data collection

Dry matter degradation, vessel pH, overflow container pH, TGP and CH_4 percentage of TGP were continuously measured throughout the experiment. On d – 11-17, a 4 ml sample was collected from the overflow containers with the pH immediately measured using a digital

pH meter (Phoenix Instrument EC-25 Ph/Conductivity Portable Meter). This sample was then acidified using 1 ml of 50% trichloroacetic acid and stored at -20℃ for VFA analysis.

Nylon bags, after collection, were rinsed with cold water, and feed residues were washed in a domestic washing machine using the cold rinse cycle without detergent (30 min) to remove loosely attached bacteria. The feed residues were then dried in a 55°C forced air oven for 48 h and weighed. Feed dry matter degradation was calculated based on the material disappearance from the nylon bags after 24 h and 48 h of incubation for concentrates and forage, respectively. Chemical composition of the dried incubation residues was determined to calculate the degradation of feed components.

Total gas production was measured using reusable polyethylene gas bags fitted with one-way valves. Bags were attached to overflow containers where the gas was collected and measured using a DC dry gas test meter (Shinagwa Corp.; Tokyo, Japan). From here, the CH_4 percentage was analysed using an infra-red ADC SB2000 wall mounted analyser (ADC Gas Analysis; Hoddeston, UK). Daily calibrations of the ADC SB2000 were conducted with a 10% CH_4 span gas. The CH_4 % and TGP were then used to calculate CH_4 (mmol/d) using the following formula:

$$CH_4(mmol\ d^{-1}) = \frac{[CH_4(\%) \times TGP\ (L\ d^{-1})] \times 0.656}{16.04} \times 1000$$

Where CH_4 % × TGP gives the volume of CH_4 (L/d), 0.656 g/L is the density of CH_4 and 16.04 g/mol is the molecular weight of CH_4, and the factor 1000 converts mol to mmol.

Chemical analysis

Feed analysis

The DM content of the feed samples was determined by drying the samples at 105 ℃ for 16 h [AOAC 26a; method 960.15]. Ash concentrations were determined through complete

combustion in a muffle furnace (Nabertherm, GmbH, Lilienthal, Germany) at 550 °C for 4 h [AOAC 26b; method 942.05]. Nitrogen concentrations of the feed were assessed using a LECO FP 528 instrument (Leco Instruments, UK, Ltd, Stockport, UK), and the resulting values were multiplied by 6.25 to determine crude protein (CP) concentrations [AOAC 26c; method 990.03]. Neutral detergent fibre (NDF) and acid detergent fibre (ADF) concentrations were determined following the method of Van Soest [27] using the ANKOM220 Fibre Analyzer (ANKOM Technology, Macedon, NY). Forage and concentrate samples were analysed for NDF with sodium sulphite, with heat stable amylase included for concentrate samples only. The starch content of feed was determined using the Megazyme Total Starch Assay Procedure (product no. K-TSTA, Megazyme International Ltd., Wicklow, Ireland). Gross energy (GE) content of the feed was determined by bomb calorimeter (Parr 1281 Bomb Calorimeter, Parr Instrument Company, Moline, IL). Ether extract (EE) of feed samples was determined using a Soxtec instrument (tecator) according to the method and light petroleum ether.

Seaweed analysis

Total soluble carbohydrates (TSC) were determined by the phenol-sulphuric acid method following the protocol as described by [28]. Briefly, 100 µL of test samples or standard (D-glucose, 50-250 mg/L) were mixed thoroughly with 100 µL of 0.8% (w/v) phenol solution and 2 ml of sulphuric acid (95–98%). The mixtures were allowed to stand for 10 min and then incubated in a water bath at 30 °C for 20 min. Absorbance was measured at 490 nm using a spectrophotometer (VICTOR® Nivo™, PerkinElmer, Pontyclun, United Kingdom), and results were expressed as mg glucose equivalents (GlcE) per g of fresh sample.

Total phenolic content (TPC) and total phlorotannin content (TPhC) were determined as described by Garcia-Vaquero [29]. Briefly, 100 µL of sample or standard (gallic acid (TPC) or phloroglucinol (TPhC) at 25–300 mg/L) were mixed with 2 mL of sodium carbonate solution (2% w/v). After 2 min, 100 µL of Folin–Ciocalteu's solution (1 M) was added, and the mixtures

were incubated in dark conditions (30 min, room temperature). Absorbance was measured at 720 nm in a spectrophotometer (VICTOR® Nivo™, PerkinElmer, Pontyclun, United Kingdom). Results of TPC and TPhC were expressed as mg gallic acid equivalents (GAE) and mg phloroglucinol equivalents (PGE) per g of fresh sample, respectively.

Total tannin contents (TTC) were determined as described by Liu [30]. A volume of 50 µL of sample or standard (catechin, 15-150 mg/L) were mixed thoroughly with 1.5 ml of methanolic vanillin solution (4% w/v) and 750 µL of 37% hydrochloric acid (HCl). The mixtures were incubated in dark conditions (20 min, room temperature) and the absorbance was read at 500 nm using in a spectrophotometer (VICTOR® Nivo™, PerkinElmer, Pontyclun, United Kingdom). Results of TTC were expressed as mg catechin equivalents (ChE) per g of fresh sample.

VFA analysis

Liquid samples, previously collected from the overflow containers, acidified, and frozen pending analysis, were thawed overnight in a fridge at 4 °C and then centrifuged at 1,800 *g* for 10 min at 4 °C. Following this, 250 µl of supernatant was diluted with 3.75 ml of distilled water and 1 ml of an internal standard solution (0.5808 g 3-methyvaleric acid in 1,000 mL of HPLC grade water). The resulting solution underwent centrifugation at 260 *g* for 5 min at room temperature and was then filtered through a syringe tip filter (polytetrafluoroethylene, 25 mm diameter, 0.45 µm) into 2 ml gas chromatography vials, with each sample analysed in duplicate. The concentration of VFA’s was determined using Scion 456-GS (Scion Instrument, Scotland, UK) fitted with a DB-FFAP capillary column (15 m × 0.53 mm: 1.00 µm, Agilent Technologies, USA). Metabolic hydrogen produced, incorporated and recovered was accounted from the VFA production based off the calculation by Ungerfeld [31] without the inclusion of H_2.

Statistical analysis

Data was checked for normality and homogeneity of variance by histograms, qq plots, and formal statistical tests as part of the UNIVARIATE procedure of SAS® studio (edition 3.81). Data was analysed using the PROC MIXED procedure, with fermentation vessel included as a random effect. The model included the fixed effects of treatment and day. Differences between treatments were determined by F-tests using Type III sums of squares. The PDIFF command incorporating the Tukey test was applied to evaluate pairwise comparisons between treatment means. Statistically significant difference was assumed at $P < 0.05$ and a tendency toward significance was assumed at $P \geq 0.05$ but $P < 0.10$.

Results

Diet degradation

The degradation of DM, OM, CP and NDF in Experiment 1 and 2 are presented in **Table 3** and **4**, respectively. In Experiment 1, DM, OM and CP degradation were reduced for treatment 0.3% relative to CTR and 0.1% treatments, while NDF degradation was reduced for treatment 0.3% versus CTR only ($P < 0.05$). In Experiment 2, DM and OM degradation was reduced for treatment 0.5% versus CTR treatment ($P < 0.05$). There was no effect of treatments on CP or NDF degradation ($P > 0.05$).

Table 3. Mean effect of treatments on nutrient degradation (g/kg DM unless stated) in Experiment 1.

Parameter	Treatments				SEM	*P*-value
	CTR	0.1%	0.2%	0.3%		
**DM (g/kg)**	866^{a}	865^{a}	858^{ab}	818^{b}	13.2	0.010
**OM**	864^{a}	863^{a}	856^{ab}	814^{b}	11.8	0.011
**CP**	884^{a}	889^{a}	878^{ab}	853^{b}	8.02	0.011
**NDF**	754^{a}	742^{ab}	720^{ab}	642^{b}	29.1	0.040

$^{a-b}$ Within each column, treatment means not sharing the same superscript differ ($P < 0.05$). CTR = 0 mg/ 20g DM of *Ascophyllum nodosum* (ASC) extract; 0.1% = 20 mg/ 20g DM of ASC extract; 0.2% = 40 mg/ 20g DM of ASC extract; 0.3% = 60 mg/ 20g DM of ASC extract. DM, Dry matter; OM, Organic matter; CP, Crude protein; NDF, Neutral detergent fibre.

Table 4. Mean effect of treatments on nutrient degradation (g/kg DM unless stated) in Experiment 2.

Parameter	Treatments				SEM	*P*-value
	CTR	0.3%	0.4%	0.5%		
**DM (g/kg)**	673^{a}	656^{ab}	657^{ab}	638^{b}	7.90	0.035
**OM**	658^{a}	639^{a}	641^{ab}	621^{b}	8.13	0.032
**CP**	933	923	916	920	9.65	0.624
**NDF**	319	315	319	291	13.8	0.434

$^{a-b}$ Within each column, treatment means not sharing the same superscript differ ($P < 0.05$). CTR = 0 mg/ 20g DM of *Ascophyllum nodosum* (ASC) extract; 0.1% = 20 mg/ 20g DM of ASC extract; 0.2% = 40 mg/ 20g DM of ASC extract; 0.3% = 60 mg/ 20g DM of ASC extract. DM, Dry matter; OM, Organic matter; CP, Crude protein; NDF, Neutral detergent fibre.

Fermentation variables

The effect of ASC extract on rumen fermentation and VFA production for Experiment 1 and 2 are presented in **Table 5** and **6,** respectively. In Experiment 1, total VFA and butyrate production were reduced for treatment 0.3% compared to all other treatments ($P < 0.05$), while

acetate production was reduced relative to treatment 0.1% only ($P < 0.05$). There was no effect of treatment on the production of propionate, iso-butyrate, valerate and iso-valerate along with acetate to propionate ratio ($P > 0.05$). Metabolic hydrogen produced was reduced for treatment 0.3% relative to both CTR and 0.1% treatment, while [H] incorporated was reduced for treatment 0.3% versus CTR only ($P < 0.05$). There was no effect of treatment on [H] recovered or vessel pH ($P > 0.05$).

In Experiment 2, there was no difference between treatments for any VFA production parameters, [H] produced or incorporated and vessel pH ($P > 0.05$). Metabolic hydrogen recovered was reduced for all three inclusion rates relative to the CTR ($P < 0.05$).

Table 5. Mean effect of treatments on gas production and rumen fermentation parameters in Experiment 1.

Parameter	Treatments				SEM	*P*-value
	CTR	0.1%	0.2%	0.3%		
TGP (L/d)	3.12^{a}	3.00^{ab}	3.05^{ab}	2.50^{b}	0.158	0.035
CH_4 (mmol/d)	14.2^{a}	12.3^{ab}	12.9^{ab}	9.3^{b}	1.13	0.029
CH_4 (mmol/g DOM)	0.84^{a}	0.73^{ab}	0.76^{ab}	0.58^{b}	0.062	0.037
Vessel pH	6.32	6.37	6.32	6.47	0.063	0.284
VFA production (mmol/d)						
TVFA	70.1^{a}	70.3^{a}	70.7^{a}	58.1^{b}	2.75	0.022
Acetate	33.6^{ab}	34.9^{a}	34.3^{ab}	27.2^{b}	1.63	0.026
Propionate	16.3	16.4	17.1	14.5	0.72	0.161
Butyrate	14.5^{a}	13.7^{a}	14.0^{a}	11.3^{b}	0.57	0.007
Iso-butyrate	0.55	0.54	0.55	0.46	0.032	0.231
Valerate	3.07	2.58	2.84	2.69	0.170	0.210
Iso-valerate	2.04	2.01	2.01	1.92	0.109	0.872
Acetate: propionate ratio	2.07	2.12	2.02	1.87	0.078	0.192
Estimated H parameters (mmol/d)						
[H] production	141.7^{a}	141.4^{a}	141.7^{a}	114.7^{b}	5.66	0.012
[H] incorporated	119.6^{a}	106.5^{ab}	115.4^{ab}	88.7^{b}	7.67	0.042
[H] recovery (%)	84.3	75.5	84.6	77.4	4.15	0.383

[a-b] Within each column, treatment means not sharing the same superscript differ ($P < 0.05$)

[1]CTR = 0 mg/ 20g DM of *Ascophyllum nodosum* (ASC) extract; 0.1% = 20 mg/ 20g DM of ASC extract; 0.2% = 40 mg/ 20g DM of ASC extract; 0.3% = 60 mg/ 20g DM of ASC extract

TGP, Total gas production; CH_4, Methane; DOM, digestible organic matter; TVFA, Total volatile fatty acids; [H], Metabolic hydrogen.

Total gas and methane parameters

Table 5 and **6** report the effect of ASC extract on TGP (L/d) and CH_4 parameters (mmol/d, and CH_4 mmol/g digestible organic matter (DOM) for Experiment 1 and 2. In

Experiment 1, inclusion of ASC extract at 0.3% resulted in a 20% reduction in TGP, 30% reduction in CH_4 (mmol/d) and 30% reduction in CH_4 (mmol/g DOM) compared to CTR ($P < 0.05$).

In Experiment 2, all three inclusion rates of ASC extract (0.3%, 0.4% and 0.5%) reduced TGP, CH_4 (mmol/d) and CH_4 (mmol/g DOM) compared to the CTR ($P < 0.05$). For TGP, there was a reduction of 21%, 24% and 20% compared to the CTR for treatments 0.3%, 0.4% and 0.5%, respectively. For CH_4 (mmol/d), there was a reduction of 26%, 28% and 22% relative to the CTR for treatments 0.3%, 0.4% and 0.5%, respectively. While for CH_4 (mmol/g DOM), there was a reduction of 28%, 27% and 21% in comparison to the CTR for treatments 0.3%, 0.4% and 0.5%, respectively.

Table 6. Mean effect of treatments on gas production and rumen fermentation parameters in Experiment 2.

Parameter	Treatments				SEM	*P*-value
	CTR	0.3%	0.4%	0.5%		
**TGP (L/d)**	2.61^{a}	2.04^{b}	1.98^{b}	2.07^{b}	0.124	0.003
**CH_4 (mmol/d)**	8.8^{a}	6.5^{b}	6.4^{b}	6.8^{b}	0.47	0.029
**CH_4 (mmol/g DOM)**	0.69^{a}	0.49^{b}	0.50^{b}	0.54^{b}	0.034	0.020
**Vessel pH**	7.09	7.06	7.06	7.10	0.023	0.554
**VFA production (mmol/d)**						
**TVFA**	46.5	46.1	45.9	51.2	2.09	0.241
**Acetate**	20.9	20.7	21.4	23.8	1.03	0.159
**Propionate**	11.4	10.9	11.4	12.7	0.63	0.292
**Butyrate**	8.10	8.38	7.82	8.87	0.410	0.329
**Iso-butyrate**	0.37	0.36	0.35	0.40	0.022	0.462
**Valerate**	3.75	3.86	3.37	3.74	0.159	0.188
**Iso-valerate**	1.77	1.71	1.62	1.72	0.084	0.648
**Acetate: propionate ratio**	1.85	1.88	1.88	1.88	0.040	0.909
**Estimated H parameters (mmol/d)**						
**[H] production**	86.0	85.9	85.3	97.3	4.28	0.182
**[H] incorporated**	77.7	67.9	67.6	78.2	3.66	0.091
**[H] recovery (%)**	90.3^{a}	78.9^{b}	79.5^{b}	78.6^{b}	2.97	0.034

[a-b] Within each column, treatment means not sharing the same superscript differ ($P < 0.05$)

[1]CTR = 0 mg/ 20g DM of *Ascophyllum nodosum* (ASC) extract; 0.1% = 20 mg/ 20g DM of ASC extract; 0.2% = 40 mg/ 20g DM of ASC extract; 0.3% = 60 mg/ 20g DM of ASC extract

TGP, Total gas production; CH_4, Methane; DOM, digestible organic matter; TVFA, Total volatile fatty acids; [H], Metabolic hydrogen.

Discussion

This study investigated the effects of an ASC extract on nutrient degradation, rumen fermentation and CH_4 production *in vitro* using the RUSITEC system. The novelty of this study lies in the use of liquid ASC extracts containing various bioactive compounds, tested at different inclusion rates. The hypothesis proposed that the inclusion of an ASC extract would reduce TGP and CH_4 production. As the findings demonstrated that the ASC extract reduced both TGP and CH_4 production, the hypothesis is accepted. In Experiment 1, the inclusion of an ASC extract at 0.3% to an 80:20 grass-to-concentrate diet reduced all nutrient degradability parameters, TVFA production, TGP and CH_4 production. In Experiment 2, when added to an 80:20 silage-to-concentrate diet, all three inclusion rates of ASC extract (0.3%, 0.4% and 0.5%) reduced TGP and CH_4 production, while 0.5% lowered DM and OM degradability.

Diet degradation

A 34% reduction in CH_4 production with the addition of an ASC extract to an 80:20 F:C diet was identified in the current study. The highest inclusion level (0.3% for a grass-based diet and 0.5% for a silage-based diet) of an ASC extract reduced degradation of DM and OM, with reductions in CP and NDF degradation in Experiment 1 (grass-based diet) only. The observed reductions in degradation parameters are likely attributable to the PT content in the seaweed extract, which effect nutrient degradability through antimicrobial activity and nutrient complex formation.

The antimicrobial properties of PT, particularly their effects on fibre-degrading bacteria [18], and their nutrient-binding abilities [32] are well-documented. In this study, the higher PT concentrations (408 g/kg DM in Experiment 1; 343 g/kg DM in Experiment 2) likely amplified these effects, reducing nutrient degradation. Similarly, Kunzel [19] reported reductions in all nutrient degradability parameters when dried ASC (5% DM) with a PT concentration of 79

g/kg DM was added to a 60:40 F:C diet. In contrast, Roskam [21] observed no effect on nutrient degradation when ASC was included at 1% DM in a 50:50 F:C diet, likely due to the much lower PT concentration (107 g/kg DM). Belanche [20] included dried ASC at 5% DM but observed reductions in N degradability only, not in fibre degradability. This can be attributed to the lower PT concentration (2.44 g/kg DM) and 50:50 F:C ratio, which affected protein-binding but not carbohydrate-binding [20]. Discrepancies among these studies can be attributed to differences in ASC inclusion level, PT concentrations and F:C ratios. Additionally, PT's show a greater affinity towards fibre-degrading bacteria in comparison to starch-degrading bacteria [18], emphasizing the diet-dependent nature of PT's impacts. These effects vary with microbial population and substrate composition, further highlighting the importance of diet composition determining PT's efficacy.

The grass-based diet in Experiment 1 showed reductions in CP and NDF degradation, while no such effects were observed with the silage-based diet in Experiment 2. This differential response highlights how PT's impacts are modulated by diet composition, with the lower NDF and higher CP content of the grass-based diet enhancing PT's nutrient-binding effects. Additionally, the rapid fermentability of grass may have increased its susceptibility to PT's antimicrobial effects. These findings highlight the diet-dependent nature of PT's impacts, as its effects vary with microbial population and substrate composition.

A reduction in nutrient degradability would result in reduced nutrient and energy availability for ruminants [19]. While a reduction in ruminal CP degradability could be compensated for by an increased post-ruminal degradation, allowing ruminally undegraded protein to be efficiently utilized in the small intestine [33], this is not the case for NDF degradability. Neutral detergent fibre in the rumen is primarily degraded by fibre-degrading microbes [34], and any reduction in its ruminal degradation is unlikely to be compensated for post-ruminally. Therefore, a reduction in NDF degradability is likely to result in a net loss of

digestible energy, potentially impacting animal performance. Optimizing PT inclusion rates is crucial to balance CH_4 mitigation with nutrient availability.

Fermentation parameters

The reduced nutrient degradation in Experiment 1 with the grass-based diet likely decreased substrate availability for fermentation, resulting in lower VFA production. Tannins can reduce the rate of carbohydrate digestion and decrease TVFA concentrations [17, 35]. In the present study, TVFA's and butyrate production were reduced at the 0.3% inclusion level compared to the control, while acetate production was reduced relative to the 0.1% treatment only. These effects align with findings by Kunzel [19] and Wang [18], who observed similar reductions in VFA's and gas production due to PT's inhibitory effects on microbial activity and nutrient degradation.

In Experiment 2, while DM and OM degradation decreased, CP and NDF degradation were unaffected. This suggests that fermentation dynamics are influenced by specific microbial populations responsible for fibre and protein degradation. Notably, NDF degradability appears to play a critical role in fermentation as Wang [18] observed that reduced aNDF degradability was linked to a reduction in total gas and TVFA production, while Belanche [20] found that reductions in N degradability alone had no effect on the rumen fermentation. These findings align with the results of the current study, where the lack of effect on NDF degradability corresponds with the absence of negative impacts on VFA production.

Metabolic hydrogen produced and incorporated decreased with the 0.3% treatment in Experiment 1, which is in line with the proposed relationship between [H] flux and CH_4 production [31]. Despite this, [H] recovery remained consistent, suggesting that the balance between [H] produced and incorporated was maintained, even though microbial activity occurred at a lower level. In Experiment 2, [H] recovered was reduced across all inclusion

levels, despite no change in [H] or VFA production. This suggests a redirection of [H] away from methanogenesis [36], potentially disrupting methanogenic activity. Previous *in vitro* work with red and brown seaweed has demonstrated this inhibition of methanogen populations [19, 37]. Therefore, it is plausible that the ASC extract used in this study impacted methanogenic activity, leading to lower [H] recovery and CH_4 production.

Rumen pH plays a vital role in maintaining a stable rumen function because of its effects on microbial fermentation and fermentation end products [38]. Rumen pH remained unaffected by treatments in both experiments, likely due to the highly buffered nature of the *in vitro* system, which is designed to prevent significant pH fluctuations. However, differences between experiments were observed, with the grass-based diet in Experiment 1 producing higher VFA concentrations and lower pH than the grass silage-based diet in Experiment 2. These variations are consistent with differences in substrate fermentability, as rapidly fermentable substrate like grass generate more VFA's and lower pH compared to less fermentable silage. Rumen pH is negatively correlated with VFA production [39], and hence the greater amount of VFA's seen in Experiment 1 coincide with the lower vessel pH values recorded in that experiment.

Total gas and methane parameters

There have been promising results involving the use of red seaweed species in reducing CH_4 emissions [40-42], however, the anti-methanogenic effect of such species can be attributed to the presence of halogenated compounds such as bromoform and dibromochloromethane [43]. The anti-methanogenic potential of brown seaweed species on the other hand, can be aligned towards the concentration of other bioactive compounds such as PT [13]. In the current study, the addition of an ASC extract reduced CH_4 production by up to 34% with mechanisms varying by diet. In Experiment 1, reductions in CH_4 were linked to decreased nutrient

degradation and VFA production, reflecting a trade-off between CH_4 mitigation and nutrient utilization. Conversely, in Experiment 2, CH_4 reductions occurred without significant effects on VFA's or nutrient degradability, suggesting alternative mechanisms were at play such as redirecting H_2 away from methanogenesis or inhibiting methanogen population.

Reductions in CH_4 production have been linked with either a reduction in overall rumen fermentation [11], and hence TVFA production, or by a shift in the VFA proportions [44]. As the acetate to propionate ratio remained unaffected in this study, this suggests that the reduction in overall rumen fermentation, rather than shifts in VFA proportions, were the primary factors reducing CH_4. Similar findings were reported by Kunzel [19], who observed reductions in rumen fermentation and CH_4 production when administering dried ASC at 5% DM *in vitro*. In contrast, studies by Belanche [20] and Roskam [21], found limited effects on rumen fermentation and CH_4 production, likely due to lower PT concentrations and ASC dosage rates, respectively.

The observed reduction in [H] recovered in Experiment 2 suggests that not all H_2 was accounted for in CH_4 or VFA end products. Since methanogens are the primary microbes responsible for converting H_2 into CH_4 during rumen fermentation [45], it is possible that PT influenced the methanogen population. Supporting this, Kunzel [19] reported a reduction in the most abundant rumen methanogen genus, *Methanobrevibacter*, with the inclusion of dried ASC *in vitro*. Similarly, Belanche [20] observed decreased methanogen and protozoal populations with dried ASC supplementation. Although the methanogen community was not directly assessed in this study, it is reasonable to suggest that methanogens were impacted by the ASC extract. This disruption would reduce the efficiency of H_2 conversion to CH_4, contributing to the observed reduction in CH_4 production. These results highlight the need to balance CH_4 mitigation with maintaining nutrient and energy availability.

Conclusion

This study demonstrates that ASC extracts can reduce TGP and CH_4 production *in vitro*, with the mode of action differing according to the basal diet. In Experiment 1, when added to a grass-based diet, all nutrient degradability parameters were reduced, which lowered TVFA and butyrate production, leading to decreased TGP and CH_4 production at 0.3% relative to CTR. In Experiment 2, when added to a silage-based diet, all ASC extract treatments reduced TGP and CH_4 production when added to a silage-based diet. Although DM and OM degradability was reduced at 0.5% treatment, there was no effect on NDF or CP degradation, or VFA production, indicating an alternative mode of action in lowering TGP and CH_4 production. Indeed, [H] recovery was lower for all ASC extract treatments relative to CTR, indicating that not all H_2 was converted to CH_4 or VFA's. Findings from this study highlight that ASC extracts can lower CH_4 production *in vitro*, supporting their potential use as a CH_4 mitigation strategy in ruminant production systems. However, the contrasting responses observed across different basal diets highlight the need for further investigation.

Acknowledgements

The authors would like to thank BioAtlantis Ltd for the support provided throughout this study, and to the laboratory staff and postgraduate students at UCD Lyons Farm for the help provided during this study.

Author Contributions

Conceptualization: Tommy Boland, Zoe McKay, Sivaprakash Balakrishnan, Kieran Guinan, J.T. O'Sullivan, Stafford Vigors.

Data curation: Aaron Casey, Alan Kelly.

Formal analysis: Aaron Casey, Alan Kelly.

Investigation: Aaron Casey.

Methodology: Aaron Casey, Alan Kelly, Zoe McKay, Marco Garcia-Vaquero, Armin Mirzapour-Koudash, Sivaprakash Balakrishnan, Kieran Guinan, J.T. O'Sullivan, Maria Markiewicz-Keszycka.

Project administration: Tommy Boland, Stafford Vigors.

Supervision: Tommy Boland, Stafford Vigors.

Writing – original draft: Aaron Casey

Writing – review & editing: Aaron Casey, Tommy Boland, Alan Kelly, Zoe McKay, Stafford Vigors.

References

1. EPA. Ireland's Provisional GreenhouseGas Emissions. 1990-2023. Environmental Protection Agency. 2024. Available from https://www.epa.ie/publications/monitoring--assessment/climate-change/air-emissions/EPA-Provisional-GHG-Report-Jul24-v6.pdf.
2. Janssen, P.H. Influence of hydrogen on rumen methane formation and fermentation balances through microbial growth kinetics and fermentation thermodynamics. Anim. Feed Sci. Technol. 2010. 160(1-2):1-22. doi:10.1016/j.anifeedsci.2010.07.002
3. Beijer, W. Methane fermentation in the rumen of cattle. Nature. 1952. 170(4327):576-577. doi:10.1038/170576a0
4. Ferry, J.G. Fundamentals of methanogenic pathways that are key to the biomethanation of complex biomass. Curr. Opin. Biotechnol. 2011. 22(3):351-357. doi:10.1016/j.copbio.2011.01.011
5. Johnson, K.A. and D.E. Johnson. Methane emissions from cattle. J. Anim. Sci. 1995. 73(8):2483-2492. doi:10.2527/1995.7382483x
6. CCAC. Carbon Budget Technical Report. In: Climate Change Advisory Council - Carbon Budget Technical Report. pp:16-18. October. 2021. Available from https://www.climatecouncil.ie/media/climatechangeadvisorycouncil/Technical%20report%20on%20carbon%20budgets%2025.10.2021.pdf.
7. McAllister, T.A. and C.J. Newbold. Redirecting rumen fermentation to reduce methanogenesis. Aust. J. Exp. Agric. 2008. 48(2). doi:10.1071/ea07218
8. Beauchemin, K.A.; M. Kreuzer; F. O'Mara, and T.A. McAllister. Nutritional management for enteric methane abatement: a review. Aust. J. Exp. Agric. 2008. 48(2). doi:10.1071/ea07199

9. Hristov, A.N.; J. Oh; J.L. Firkins; J. Dijkstra; E. Kebreab; G. Waghorn; H.P. Makkar; A.T. Adesogan; W. Yang; C. Lee; P.J. Gerber; B. Henderson, and J.M. Tricarico. Special topics--Mitigation of methane and nitrous oxide emissions from animal operations: I. A review of enteric methane mitigation options. J. Anim. Sci. 2013. 91(11):5045-69. doi:10.2527/jas.2013-6583

10. Hristov, A.N.; A. Melgar; D. Wasson, and C. Arndt. Symposium review: Effective nutritional strategies to mitigate enteric methane in dairy cattle. J. Dairy Sci. 2022. 105(10):8543-8557. doi:10.3168/jds.2021-21398

11. Beauchemin, K.A.; T.A. McAllister, and S.M. McGinn. Dietary mitigation of enteric methane from cattle. CAB Rev. Perspect. Agric. Vet. Sci. Nutr. Nat. Resour. 2009. 2009:1-18. doi:10.1079/pavsnnr20094035

12. Beauchemin, K.A.; E.M. Ungerfeld; R.J. Eckard, and M. Wang. Review: Fifty years of research on rumen methanogenesis: lessons learned and future challenges for mitigation. Animal. 2020. 14(S1):s2-s16. doi:10.1017/S1751731119003100

13. Abbott, D.W.; I.M. Aasen; K.A. Beauchemin; F. Grondahl; R. Gruninger; M. Hayes; S. Huws; D.A. Kenny; S.J. Krizsan; S.F. Kirwan; V. Lind; U. Meyer; M. Ramin; K. Theodoridou; D. von Soosten; P.J. Walsh; S. Waters, and X. Xing. Seaweed and Seaweed Bioactives for Mitigation of Enteric Methane: Challenges and Opportunities. Animals. 2020. 10(12). doi:10.3390/ani10122432

14. Machado, L.; M. Magnusson; N.A. Paul; R. de Nys, and N. Tomkins. Effects of marine and freshwater macroalgae on in vitro total gas and methane production. PloS ONE. 2014. 9(1):e85289. doi:10.1371/journal.pone.0085289

15. Roque, B.M.; M. Venegas; R.D. Kinley; R. de Nys; T.L. Duarte; X. Yang, and E. Kebreab. Red seaweed (Asparagopsis taxiformis) supplementation reduces enteric

methane by over 80 percent in beef steers. PloS ONE. 2021. 16(3):e0247820. doi:10.1371/journal.pone.0247820

16. Ragan, M.A. Phlorotannins, brown algal polyphenols. Prog. Phycol. Res. 1986. 4:129-241.

17. Patra, A.K. and J. Saxena. Exploitation of dietary tannins to improve rumen metabolism and ruminant nutrition. J. Sci. Food Agric. 2011. 91(1):24-37. doi:10.1002/jsfa.4152

18. Wang, Y.; Z. Xu; S.J. Bach, and T.A. McAllister. Effects of phlorotannins from Ascophyllum nodosum (brown seaweed) on in vitro ruminal digestion of mixed forage or barley grain. Anim. Feed Sci. Technol. 2008. 145(1-4):375-395. doi:10.1016/j.anifeedsci.2007.03.013

19. Kunzel, S.; T. Yergaliyev; K.J. Wild; H. Philippi; A.H. Petursdottir; H. Gunnlaugsdottir; C.K. Reynolds; D.J. Humphries; A. Camarinha-Silva, and M. Rodehutscord. Methane Reduction Potential of Brown Seaweeds and Their Influence on Nutrient Degradation and Microbiota Composition in a Rumen Simulation Technique. Front. Microbiol. 2022. 13:889618. doi:10.3389/fmicb.2022.889618

20. Belanche, A.; E. Jones; I. Parveen, and C.J. Newbold. A Metagenomics Approach to Evaluate the Impact of Dietary Supplementation with Ascophyllum nodosum or Laminaria digitata on Rumen Function in Rusitec Fermenters. Front. Microbiol. 2016. 7:299. doi:10.3389/fmicb.2016.00299

21. Roskam, E.; S.F. Kirwan; D.A. Kenny; C. O’Donnell; V. O’Flaherty; M. Hayes, and S.M. Waters. Effect of brown and green seaweeds on diet digestibility, ruminal fermentation patterns and enteric methane emissions using the rumen simulation technique. Front. Anim. Sci. 2022. 3. doi:10.3389/fanim.2022.1021631

22. Czerkawski, J.W. and G. Breckenridge. Design and development of a long-term rumen simulation technique (Rusitec). Br. J. Nutr. 1977. 38(3):371-84. doi:10.1079/bjn19770102

23. Hristov, A.N.; C. Lee; R. Hristova; P. Huhtanen, and J.L. Firkins. A meta-analysis of variability in continuous-culture ruminal fermentation and digestibility data. J. Dairy Sci. 2012. 95(9):5299-5307. doi:10.3168/jds.2012-5533

24. Jaurena, G.; J.M. Moorby, and D.R. Davies. Efficiency of microbial protein synthesis on red clover and ryegrass silages supplemented with barley by rumen simulation technique (RUSITEC). Anim. Feed Sci. Technol. 2005. 118(1-2):79-91. doi:10.1016/j.anifeedsci.2004.09.008

25. McDougall, E.I. Studies on ruminant saliva. 1. The composition and output of sheep's saliva. Biochem. J. 1948. 43(1):99-109. doi:10.1042/bj0430099

26. AOAC. Official Methods of Analysis. 18th Ed. Gaithersburg (MD): AOAC International; 2005.

27. Van Soest, P.v.; J.B. Robertson, and B.A. Lewis. Methods for dietary fiber, neutral detergent fiber, and nonstarch polysaccharides in relation to animal nutrition. J. Dairy Sci. 1991. 74(10):3583-3597. doi:10.3168/JDS.s0022-0302(91)78551-2

28. Brummer, Y. and S.W. Cui. Understanding carbohydrate analysis. CRC press. 2005.1-38. doi:10.1201/9780203485286

29. Garcia-Vaquero, M.; R. Ravindran; O. Walsh; J. O'Doherty; A.K. Jaiswal; B.K. Tiwari, and G. Rajauria. Evaluation of ultrasound, microwave, ultrasound–microwave, hydrothermal and high pressure assisted extraction technologies for the recovery of phytochemicals and antioxidants from brown macroalgae. Mar. Drugs. 2021. 19(6):309. doi:10.3390/md19060309

30. Liu, S.-C.; J.-T. Lin; C.-K. Wang; H.-Y. Chen, and D.-J. Yang. Antioxidant properties of various solvent extracts from lychee (Litchi chinenesis Sonn.) flowers. Food Chem. 2009. 114(2):577-581. doi:10.1016/j.foodchem.2008.09.088
31. Ungerfeld, E.M. Shifts in metabolic hydrogen sinks in the methanogenesis-inhibited ruminal fermentation: a meta-analysis. Front. Microbiol. 2015. 6:37. doi:10.3389/fmicb.2015.00037
32. McAllister, T.; H. Bae; G. Jones, and K.-J. Cheng. Microbial attachment and feed digestion in the rumen. J. Anim. Sci. 1994. 72(11):3004-3018. doi:10.2527/1994.72113004x
33. Bach, A. Nitrogen metabolism in the rumen. J. Dairy Sci. 2005. doi:10.3168/jds.S0022-0302(05)73133-7
34. Weimer, P.J. Degradation of cellulose and hemicellulose by ruminal microorganisms. Microorganisms. 2022. 10(12):2345. doi:10.3390/microorganisms10122345
35. Makkar, H.P.; M. Blümmel, and K. Becker. In vitro effects of and interactions between tannins and saponins and fate of tannins in the rumen. J. Sci. Food Agric. 1995. 69(4):481-493. doi:10.1002/jsfa.2740690413
36. Guyader, J.; E.M. Ungerfeld, and K.A. Beauchemin. Redirection of Metabolic Hydrogen by Inhibiting Methanogenesis in the Rumen Simulation Technique (RUSITEC). Front. Microbiol. 2017. 8:393. doi:10.3389/fmicb.2017.00393
37. O'Hara, E.; S.A. Terry; P. Moote; K.A. Beauchemin; T.A. McAllister; D.W. Abbott, and R.J. Gruninger. Comparative analysis of macroalgae supplementation on the rumen microbial community: Asparagopsis taxiformis inhibits major ruminal methanogenic, fibrolytic, and volatile fatty acid-producing microbes in vitro. Front. Microbiol. 2023. 14:1104667. doi:10.3389/fmicb.2023.1104667

38. Ellis, J.L.; J. Dijkstra; E. Kebreab; A. Bannink; N.E. Odongo; B.W. McBride, and J. France. Aspects of rumen microbiology central to mechanistic modelling of methane production in cattle. J. Agric. Sci. 2008. 146(2):213-233. doi:10.1017/s0021859608007752

39. Dijkstra, J.; J.L. Ellis; E. Kebreab; A.B. Strathe; S. López; J. France, and A. Bannink. Ruminal pH regulation and nutritional consequences of low pH. Anim. Feed Sci. Technol. 2012. 172(1-2):22-33. doi:10.1016/j.anifeedsci.2011.12.005

40. Kinley, R.D.; R. de Nys; M.J. Vucko; L. Machado, and N.W. Tomkins. The red macroalgae Asparagopsis taxiformis is a potent natural antimethanogenic that reduces methane production during in vitro fermentation with rumen fluid. Anim. Prod. Sci. 2016. 56(3). doi:10.1071/an15576

41. Machado, L.; N. Tomkins; M. Magnusson; D.J. Midgley; R. de Nys, and C.P. Rosewarne. In Vitro Response of Rumen Microbiota to the Antimethanogenic Red Macroalga Asparagopsis taxiformis. Microb. Ecol. 2018. 75(3):811-818. doi:10.1007/s00248-017-1086-8

42. Stefenoni, H.A.; S.E. Raisanen; S.F. Cueva; D.E. Wasson; C.F.A. Lage; A. Melgar; M.E. Fetter; P. Smith; M. Hennessy; B. Vecchiarelli; J. Bender; D. Pitta; C.L. Cantrell; C. Yarish, and A.N. Hristov. Effects of the macroalga Asparagopsis taxiformis and oregano leaves on methane emission, rumen fermentation, and lactational performance of dairy cows. J. Dairy Sci. 2021. 104(4):4157-4173. doi:10.3168/jds.2020-19686

43. Machado, L.; M. Magnusson; N.A. Paul; R. Kinley; R. de Nys, and N. Tomkins. Identification of bioactives from the red seaweed Asparagopsis taxiformis that promote antimethanogenic activity in vitro. J. Appl. Phycol. 2016. 28:3117-3126. doi:10.1007/s10811-016-0830-7

44. Wang, K.; B. Xiong, and X. Zhao. Could propionate formation be used to reduce enteric methane emission in ruminants? Sci. Total Environ. 2023. 855:158867. doi:10.1016/j.scitotenv.2022.158867

45. Janssen, P.H. and M. Kirs. Structure of the archaeal community of the rumen. Appl. Environ. Microbiol. 2008. 74(12):3619-25. doi:10.1128/AEM.02812-07
