# Supplementary material for: Evaluation of Ascophyllum nodosum extract supplementation on feed degradability, ruminal fermentation and methane production using the rumen simulation technique (RUSITEC)
Source: PLoS One. 2026 Mar 25;21(3):e0344019. doi: 10.1371/journal.pone.0344019 (PMC13016304; doi:10.1371/journal.pone.0344019)
Supplement: Attachment — Submitted filename: PONE-D-25-49338_Comment.pdf [file pone.0344019.s003.pdf]

Manuscript Number: PONE-D-25-49338

Title: Evaluating the effect of an Ascophyllum nodosum extract on diet degradability, ruminal fermentation and methane production using the rumen simulation technique (RUSITEC)

General Comments

This work has potential value for publication in *PLOS ONE*, provided that several key issues are addressed. The manuscript would benefit from clearer hypotheses, improved description of experimental design, and enhanced discussion of biological implications. Specific comments by section are outlined below.

Specific Comments

Abstract

Line 27-28: Summarize results concisely. Quantify methane reduction (e.g., "Methane decreased by 12–18% relative to control"). Conclude with practical significance (e.g., "These results indicate that *A. nodosum* extract may serve as a natural methane mitigation strategy under controlled fermentation conditions.")

Introduction

Line 54-56: The text states that phlorotannins affect the activity of fiber-degrading microorganisms. However, in ruminants, these fiber-degrading microbes play a crucial role in feed digestion and overall nutrient utilization. Include discussion of phlorotannins and their possible role in modulating rumen fermentation.

Line 73-76: Add a clearly stated hypothesis. For instance, "A. nodosum extract would reduce methane emissions without compromising fermentation efficiency."

Provide rationale for selecting grass- vs silage-based diets to highlight differences in fermentability and baseline methane output.

Results

Present summarized graphs (e.g., bar charts for methane and total gas production) for improved readability.

Report % change or relative differences for methane reduction.

Discuss whether methane reduction corresponds to lower total gas or degradability.

Present molar proportions of VFAs (acetate, propionate, butyrate) to support interpretation of fermentation patterns.

Ensure that all tables include p-values or superscripts for statistical differences.

Discussion

Line 287-292: Begin with a brief summary of main findings before interpretation.

Line 401: Discuss potential mechanisms — e.g., phlorotannin effects on methanogens, hydrogen flow, or protozoal populations.

Acknowledge the limitation of lacking microbial community data.

Include updated literature (2020–2024) on seaweed extracts and methane mitigation.

Discuss practical application and potential use in *in vivo* feeding systems.

Conclusion

Conclude with future research directions (e.g., microbiome analysis, long-term feeding trials).

Table

The ash content values in Table 1 (Chemical composition) should be rechecked for accuracy.
