# Supplementary material for: Evaluation of Ascophyllum nodosum extract supplementation on feed degradability, ruminal fermentation and methane production using the rumen simulation technique (RUSITEC)
Source: PLoS One. 2026 Mar 25;21(3):e0344019. doi: 10.1371/journal.pone.0344019 (PMC13016304; doi:10.1371/journal.pone.0344019)
Supplement: Attachment — Submitted filename: Plos_Response.docx [file pone.0344019.s005.docx]

Please see responses to the below questions. We appreciate the time taken to review the manuscript

Stafford Vigors

1. We note that your author list was updated during the revision process. In order to add or remove authors or update the order of the author byline after initial submission, we ask that authors complete an Authorship Change Request form. You may review our full authorship change policy and download the Authorship Change Request form here: <https://journals.plos.org/plosone/s/authorship#loc-authorship-changes>.

   Please fill out all required sections of the form. If you are adding or removing more than 2 authors, you can complete multiple forms and submit as many forms as needed to reflect the updates. Please return the form(s) as an attachment by emailing [plosone@plos.org](mailto:plosone@plos.org) or by uploading it as a submission file labeled with the file type ‘Other’. The form will be reviewed by PLOS staff for approval. Should the changes be approved, we will require written confirmation from all authors confirming that they approve of the changes. Please note that if your manuscript is accepted, we will not be able to complete the publication process without the completed form.

   **Response:** I have responded through email regarding the approval for all authors regarding the change. I have uploaded the response email and the author change request form

2. "We note that your Data Availability Statement is currently as follows: "All relevant data are within the manuscript and its Supporting Information files"

Please confirm at this time whether or not your submission contains all raw data required to replicate the results of your study. Authors must share the “minimal data set” for their submission. PLOS defines the minimal data set to consist of the data required to replicate all study findings reported in the article, as well as related metadata and methods (<https://journals.plos.org/plosone/s/data-availability#loc-minimal-data-set-definition>).

For example, authors should submit the following data:
- The values behind the means, standard deviations and other measures reported;
- The values used to build graphs;
- The points extracted from images for analysis.

Authors do not need to submit their entire data set if only a portion of the data was used in the reported study.

If your submission does not contain these data, please either upload them as Supporting Information files or deposit them to a stable, public repository and provide us with the relevant URLs, DOIs, or accession numbers. For a list of recommended repositories, please see <https://journals.plos.org/plosone/s/recommended-repositories>.

If there are ethical or legal restrictions on sharing a de-identified data set, please explain them in detail (e.g., data contain potentially sensitive information, data are owned by a third-party organization, etc.) and who has imposed them (e.g., an ethics committee). Please also provide contact information for a data access committee, ethics committee, or other institutional body to which data requests may be sent. If data are owned by a third party, please indicate how others may request data access."

**Response:** This file has been uploaded


3. We note that the grant information you provided in the ‘Funding Information’ and ‘Financial Disclosure’ sections do not match.
When you resubmit, please ensure that you provide the correct grant numbers for the awards you received for your study.

In addition, please state what role the funders took in the study. If the funders had no role, please state: "The funders had no role in study design, data collection and analysis, decision to publish, or preparation of the manuscript."

**Response:** Funding information has now been included

Kindly include this amended Funding disclosure statement in your cover letter; we will change the online submission form on your behalf.


**Response:** Funding information has now been included on the online system
